# Novel Clones of *Streptococcus pneumoniae* Causing Invasive Disease in Malaysia

**DOI:** 10.1371/journal.pone.0097912

**Published:** 2014-06-18

**Authors:** Johanna M. Jefferies, Mohd Yasim Mohd Yusof, Shamala Devi Sekaran, Stuart C. Clarke

**Affiliations:** 1 Faculty of Medicine and Institute of Life Sciences, University of Southampton, Southampton, United Kingdom; 2 NIHR Southampton Respiratory Biomedical Research Unit, Southampton, United Kingdom; 3 Department of Medical Microbiology, Faculty of Medicine, University of Malaya, Kuala Lumpur, Malaysia; 4 Public Health England, Southampton, United Kingdom; 5 University of Southampton Malaysia Campus, Nusajaya, Malaysia; Instituto Butantan, Brazil

## Abstract

Although *Streptococcus pneumoniae* is a leading cause of childhood disease in South East Asia, little has previously been reported regarding the epidemiology of invasive pneumococcal disease in Malaysia and very few studies have explored pneumococcal epidemiology using multilocus sequence typing (MLST). Here we describe serotype, multilocus sequence type (ST), and penicillin susceptibility of thirty pneumococcal invasive disease isolates received by the University of Malaya Medical Centre between February 2000 and January 2007 and relate this to the serotypes included in current pneumococcal conjugate vaccines. A high level of diversity was observed; fourteen serotypes and 26 sequence types (ST), (11 of which were not previously described) were detected from 30 isolates. Penicillin non-susceptible pneumococci accounted for 33% of isolates. The extent of molecular heterogeneity within carried and disease-causing Malaysian pneumococci remains unknown. Larger surveillance and epidemiological studies are now required in this region to provide robust evidence on which to base future vaccine policy.

## Introduction

*Streptococcus pneumoniae* (the pneumococcus) remains a leading cause of pneumonia and invasive disease worldwide and cause significant morbidity and mortality, particularly in young children and the elderly. Pneumococci can be divided into 94 serotypes on the basis of their reactions with serum antibodies [1]–[4] and further differentiated into more than 8900 clonal types by multi locus sequence typing (MLST). Although the population of *S. pneumoniae* is very diverse, most disease worldwide is caused by around 10 serotypes [5]. There is little recent data on the epidemiology of invasive pneumococcal disease (IPD) in Malaysia; the majority of the few studies published to date have addressed serotype and/or antibiotic susceptibility [6]–[11]. A study describing clinical syndromes associated with childhood IPD during 1994–2000 was published in 2007, but does not include serotype information [12].

The heptavalent conjugate vaccine (Prevnar, PCV7, Pfizer) is effective against seven serotypes (4, 6B, 9V, 14, 18C, 19F and 23F) responsible for much paediatric IPD in many western countries and elsewhere [13], [14]. PCV7 was licensed in Malaysia in 2006, and 13-valent vaccine (Prevnar-13, PCV13, Pfizer) containing additional polysaccharides against serotypes 1, 3, 5, 7F, 18F and 19A was licensed in 2010. In addition, a 10-valent vaccine in which pneumococcal polysaccharides are conjugated to a *Haemophilus influenzae* surface protein has recently been licensed (PHiD-CV, Synflorix, GSK). However, no pneumococcal conjugate vaccine (PCV) is currently included in the Malaysian national immunisation programme (NIP).

As the distribution of pneumococcal serotypes differs between countries [15], [16], it is important to characterise the population of circulating pneumococci in specific countries in order to estimate the coverage of any particular serotype-specific pneumococcal vaccine. Gathering molecular typing data enables full characterisation of strains and facilitates comparison of clonal complexes internationally. Surveillance of antimicrobial resistance by the Asian Network for Surveillance of Resistant Pathogens (ANSORP) study group [17]–[19] has reported a rise in prevalence rates of antibiotic-resistant and non-susceptible *S. pneumoniae* isolated from carriage and disease in many Asian countries and details a rapid increase in penicillin resistant pneumococci in Malaysia between 1996 and 2001 [18].

We present results from a single centre study in which the majority of pneumococci were bloodstream isolates and demonstrate the need for larger scale surveillance studies to characterise circulating pneumococcal isolates causing both invasive and non-invasive disease within Malaysia. Even with small numbers we show that pneumococcal multi-locus sequence types (ST) causing disease in this study are heterogeneous and differ from those ST commonly observed in the USA and Europe. We also report the presence of 11 novel pneumococcal ST circulating in Kuala Lumpur; these ST have not been reported outside Malaysia at the time of writing.

## Materials and Methods

The isolates used in this study were collected at University Malaya Medical Centre (UMMC), Kuala Lumpur, Malaysia. UMMC is a large (>1200 beds), multi-disciplinary, city hospital serving Kuala Lumpur and the state of Selangor, which are the most economically developed areas of Malaysia. Individuals using the services of UMMC are mainly those from lower-middle and low-income groups due to the presence of a number of private medical centres in the area, serving mainly the upper-middle and upper income groups.

IPD was defined according to the Centers for Disease Control definition as isolation of *S. pneumoniae* from a normally sterile body site (e.g., blood, cerebrospinal fluid, or, less commonly, joint, pleural or pericardial fluid). Isolates were originally isolated from culture of invasive site clinical specimens including blood, cerebrospinal fluid (CSF) and pleural fluid, by culturing on 5% horse blood agar and incubation at 37°C in the presence of 5% CO_2_ for 12–15 hours. Alpha-haemolytic colonies were sub-cultured and confirmed as *S. pneumoniae* by their colony morphology, inability to produce catalase, ethylhydrocupreine hydrochloride (optochin) sensitivity and bile-solubility. Single colony picks were stored at −80°C in BHI broth supplemented with 10% (v/v) glycerol. The thirty pneumococcal isolates included in the study represent all viable after retrieval from ultra-cold storage from a total of 57 IPD isolates processed by UMMC during the period February 2000 to January 2007.

For this study, pneumococci were sub-cultured from frozen stocks onto 5% horse blood Columbia agar in the presence of an optochin disc. Serotyping was performed by traditional phenotypic (serological) methods and confirmed by multiplex-PCR (MP-PCR) [20].

Genotyping was performed using multi-locus sequence typing (MLST) by Qiagen Genomic Services (Hilden, Germany [21]. Briefly, ∼500 bp fragments of seven housekeeping genes were produced, compared to a database of reference sequences (http://spneumoniae.mlst.net/) and assigned an allele number. A sequence type (ST) was then assigned to each unique combination of seven allele numbers. Previously unreported alleles were assigned a new allele number by submitting raw sequence data for forward and backward DNA strands to the database curator. New combinations of previously reported alleles were assigned a new ST by the curator on submission of serotype and other isolate-specific metadata. All previously unreported STs identified in this study were deposited in the MLST database. The isolate relationships between ST were defined using eBURST version 3 [22]. Clonal Complexes (CC) were analysed in order to understand relationships between pneumococci and for comparison with pneumococci reported form elsewhere. CC were defined as groups of ST sharing six or more identical housekeeping alleles. Susceptibility to penicillin and erythromycin were determined by the E-test method [23] and susceptibility reported according to British Society for Antimicrobial Chemotherapy (BSAC) breakpoints; Penicillin S = <0.06 mg/L, I = 0.12–1 mg/L and R = >2 mg/L. Erythromcin breakpoint; R = >0.5 mg/L [24].

## Results

Of the 30 IPD isolates included in the study, eleven were from children aged less than 15 years and seventeen were from adults. No date of birth or age was recorded for the remaining two isolates. (Table 1). Information regarding the PCV vaccination status of patients was not available. Twenty-six isolates were from blood, two from cerebrospinal fluid (CSF) and two from pleural fluid. Fifteen different serotypes were detected among the 30 isolates (Table 1). Of these serotypes 19F was the most common (n = 5, 16.7%) followed by 4, 14, 19A and 23F and (n = 3, 10%) each. Twenty-six different ST, were observed among the 30 isolates (Table 1), 11 of which (49%) were novel at the time of analysis. When compared against the ST deposited in the *S. pneumoniae* MLST database (http://spneumoniae.mlst.net/) interrogated 26^th^ July 2013, all of the 15 non-novel ST in this study had previously been observed with the same serotype as that detected here.

**Table 1:** Isolates used in this study.

studyID	Specimendate	Age (yr)	Source	Serotype	ST	PCV7	PHiD-CV	PCV13
25	14/10/02	<1 mo	Blood	3	**3798**	No	Yes	Yes
80A	21/9/03	6 mo	Blood	6A	473	No	No	No
9	25/7/02	1	Blood	15A	**3801**	No	No	No
74A	18/9/06	2	Blood	9V	**4128**	Yes	Yes	Yes
64	05/11/03	4	Blood	11A	62	No	No	No
R98	07/11/00	6	CSF	23F	83	Yes	Yes	Yes
57	31/12/01	6	Blood	19F	236	Yes	Yes	Yes
91	11/02/03	9	Blood	6A	**3802**	No	No	No
87A	18/10/06	10	Blood	19F	81	Yes	Yes	Yes
43A	04/03/06	13	Blood	23F	271	Yes	Yes	Yes
70A	13/8/06	14	Blood	23F	**3800**	Yes	Yes	Yes
R48	03/07/00	20	Pleural fluid	19F	236	Yes	Yes	Yes
29	24/10/02	20	Blood	4	4127	Yes	Yes	Yes
S64	15/3/00	23	Blood	18C	113	n/a	n/a	n/a
63	05/09/03	25	Blood	16F	**3799**	No	No	No
27A	03/07/06	30	Blood	34	**3783**	No	No	No
110A	17/1/07	36	Blood	19F	236	Yes	No	Yes
17A	20/12/06	43	Blood	19A	**3781**	No	No	Yes
58A	14/7/06	50	Blood	19A	199	No	No	Yes
59A	14/7/06	50	Pleural fluid	19A	199	No	No	Yes
33A	20/3/06	52	Blood	3	458	No	No	Yes
33	07/11/02	53	Blood	19F	81	Yes	Yes	Yes
12	13/9/02	62	Blood	6B	**3797**	Yes	Yes	Yes
23	07/10/02	72	Blood	9V	162	Yes	Yes	Yes
60A	23/7/06	73	Blood	14	124	Yes	Yes	Yes
66	16/5/03	74	Blood	4	**3784**	Yes	Yes	Yes
16	22/02/2002	87	Blood	14	156	Yes	Yes	Yes
93A	11/09/06	88	Blood	14	200	Yes	Yes	Yes
18A	20/2/06	unknown	Blood	4	2213	Yes	Yes	Yes
23A	03/03/06	unknown	CSF	9N	**3782**	n/a	n/a	n/a
**Percent PCV coverage(assuming no cross protection between serotypes within a serogroup).**						57%	57%	73%

Newly-assigned ST are shown in bold typeface.

When relationships between ST were analysed using eBURST [22], we observed 16 clonal complexes (CC), 14 of which were able to be assigned a founder ST and 6 singleton ST (Figure 1). Of the 11 ST that were reported for the first time during this study, six (ST 3782, 3797, 3799, 3801, 3802 and 4128) were singleton ST, that is they did not share 6 or more housekeeping alleles with any other pneumococcus registered on the database at the time of writing (last accessed 26.07.2013). The remaining five newly assigned ST belong to five different CCs (Supplementary Figures S1–S14).

**Figure 1:**
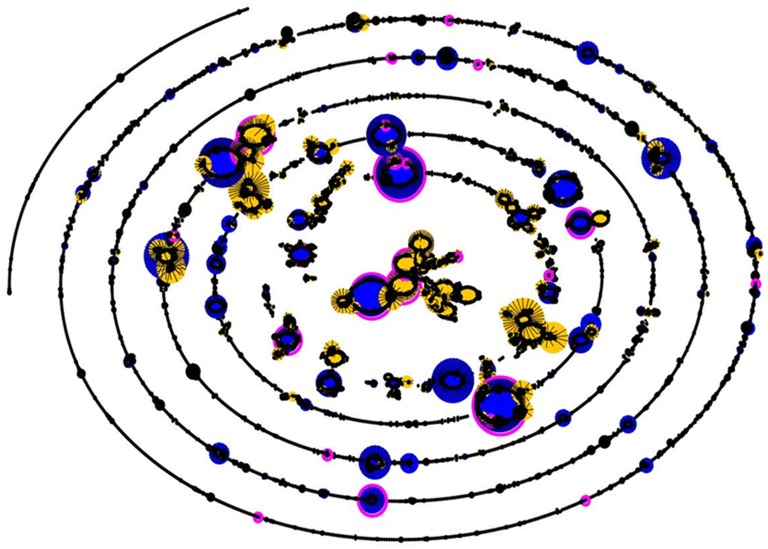
Comparative eBurst analysis of study isolates against the *S. pneumoniae* MLST database, www.mlst.net (accessed 30.07.2013). Each black dot represents one ST. Dot sizes represent the number of isolates of a given ST. Lines join STs which share 6 of 7 loci. Blue dots are the estimated founders of CCs and yellow dots sub-founders. Pink circles show STs which were observed in this study, these are also shown in text.

Ten isolates (33%) were penicillin-non-susceptible according to BSAC breakpoints, MICs ranged from 0.003 to 1.000 mg/L. None of the isolates in this study demonstrated full resistance to penicillin as defined by an MIC of >1 mg/L. Penicillin non-susceptible strains accounted for 53% (9/17) of PCV7 serotypes, non-susceptibility was confined to serotypes 14 (ST200, ST156), 19F (ST236, ST81) and 23F (ST83, ST271, ST3800) which are all included in PCV7, PHID-CV and PCV13. All but one of the penicillin non-susceptible strains were also resistant to erythromycin.

Seventeen (57%) of the isolates were of serotypes included in PCV7, three isolates were of vaccine-related serotype 19A. There was no increase in coverage with PHID-CV which includes serotypes 1, 5, and 7F in addition to those serotypes included in PCV7. PCV13, which further includes serotypes 3, 19A and 18F has coverage of 22 (73%) of the isolates included in this study.

## Discussion

This study is among the first to characterise invasive pneumococcal isolates from Malaysia by both serotype and ST. Although we characterised only a relatively small number of isolates from one centre, we can demonstrate the presence of previously unreported ST in Malaysia. Of the 26 different ST identified, nearly half (n = 11) were novel of these newly-assigned ST, five were novel combinations of alleles, which may have arisen from recombination events and six included novel alleles not previously observed (3 strains had novel *gdh* alleles and 3 strains novel *xpt* alleles). The majority of ST observed in our study mapped to the main large clonal complexes when analysed by eBURST demonstrating that globally prevalent clones are represented in our dataset (Figure 1); however, the presence of a relatively large number of newly recognised ST in a small sample may suggest that the circulating population of pneumococci in Malaysia differs from those regions such as the USA and Europe which contribute more heavily to the database.

In our collection of isolates, serotype 19F was most common, which is in agreement with other recent reports from Malaysia and other South East Asian countries [25] that 19F is among the most common serotypes. Other common serotypes being 19A, 14 and 6B [10], [26], [27] This observation also agrees with serotypes reported from other with data from Association of South East Asian Nations (ASEAN) isolates deposited on the MLST database (23F n = 51 (14%), 19A n = 44 (12%), 19F n = 40 (11%)). In this study ST236 contributed to the prevalence of serotype 19F (Table 1), this profile is that of the internationally disseminated multiply resistant clone known as Taiwan^19F^-14 (ST236) [28] which is common in many parts of the world, including South East Asia, however isolates in our study were penicillin non-susceptible, erythromycin-resistant isolates. We also observed three isolates of the vaccine-related serotype 19A. This serotype has been observed to increase in prevalence in several countries following widespread use of PCV7, although there is some evidence for a modest cross-protective effect from the 19F component [29].

Of the ST we observed, four ST (ST 81, 83, 236, 458) have also been deposited on the database from other areas of Malaysia and since our analysis, ST3781 has been observed in two further Malaysian isolates and ST 3784 has been observed in India. A further five STs (ST 156, 236, 271, 458 and 4127) have been recorded from Singapore, Thailand or Vietnam.

ST81, 236 and 271 have been observed in Thailand [30] and in Singapore (ST81, 156, 199, 200, 236, 458, 3781) [31], [32]. The most commonly deposited ST on the MLST database from ASEAN countries (those occurring with a frequency of five isolates or more) are shown in Table 2. These ST are associated with serogroups 15, 19 and 23; the latter two serogroups also rank highly among the 30 isolates examined here. It is not appropriate to make comparisons regarding serotype-ST associations between countries based on this data due to the way in which the MLST database is compiled; some researchers deposit only new STs, resulting in under-representation whereas others may upload data for all isolates in a collection, leading to over representation of clones common in particular collections.

**Table 2:** Most commonly deposited ST from ASEAN countries.

ST	Indonesia	Malaysia	Philippines	Singapore	Thailand	Vietnam	n =	PercentASEANisolates*	Serotypes
81		3		4	5	28	40	11%	19F, 23F, 23, NT
283						10	10	3%	19F
236		4				5	9	3%	19F (19)
95		1			7		8	2%	19A
320		5					5	1%	19A
1961						5	5	1%	15
5651					5		5	1%	19A
									
Total ASEAN*isolates per country	3	64	11	62	138	76	354		

*Excludes 671 isolates from the Maela Refugee Camp, Tak Province, Thailand.

The proportion of pneumococcal isolates in this study that would be covered by conjugate vaccines ranged from 57% for PCV7 and PHID-CV to 73% for PCV13. If cross protection against 6A is assumed this rises to 63% and 80% respectively. PCV7 was originally licensed on the ability to prevent invasive disease [33] but has also proved effective against pneumonia [34], [35] and otitis media [36] as well as eliciting considerable herd immunity [13]. PHID-CV has been shown to protect against AOM caused by *S. pneumoniae* and non-typeable *H. influenzae* [37].

Although PCV13 and PHiD-CV are indicated for prevention of invasive and non-invasive forms of pneumococcal disease^,^ [30], it is important to note that the isolates included in this study represent only IPD, were overwhelmingly from blood-stream isolates and were collected at one hospital during 2000–2007. While it is expected that use of PCVs in Malaysia would successfully reduce all forms of IPD caused by vaccine-serotypes, conclusions regarding vaccine coverage for meningitis and other forms of IPD cannot be directly drawn from this study. We therefore advocate further studies to investigate the serotype distribution, molecular epidemiology and antibiotic susceptibility of pneumococci causing other types of pneumococcal disease in Malaysia.

No penicillin resistant pneumococci were identified in this study, however 100% of penicillin-non susceptible isolates were of serotypes included in all three PCV. We cannot conclude that this level of coverage against penicillin non-susceptible pneumococci would be observed in other samples of disease-causing Malaysian pneumococci. It is also not appropriate to apply vaccine coverage estimates from this study to the wider Malaysian population.

As in many other countries worldwide, the prevalence of antibiotic-resistant pneumococci has been increasing in South East Asia over recent decades. The ANSORP Study Group has performed several surveillance studies designed to assess antimicrobial resistance of pneumococci in 12 South East Asian countries, including Malaysia, the results of which show that the prevalence of penicillin resistance among clinical isolates of *S. pneumoniae* has increased sharply from 3% during the period September 1996 to June 1997 to 29.5% in the period January 2000 to June 2001 [18]. More recent data from UMMC reported penicillin resistance among Malaysian clinical pneumococcal isolates to be 29.1%, with penicillin-non-susceptible isolates making up 50.3%, that study included pneumococcal isolates from both invasive and non-invasive disease [27]. In our study however, the proportion of penicillin-non-susceptible isolates was 33%. None of the isolates in our study exhibited an MIC of more than 1.0 mg/L; therefore 0% of our isolates were fully resistant to penicillin. Methods varied between the studies; we used E-test rather than the disc-diffusion method reported by Song et al. Both studies included small numbers of isolates (here n = 30, ANSORP n = 44). Small study size is likely to account for the differences in penicillin susceptibility we observed. Almost thirty percent of all the isolates included in this study (n = 9) including 90% of the penicillin-non-susceptible pneumococci were also resistant to erythromycin, with MIC’s ranging from 2 mg/L to more than 256 mg/L, this is in accordance with the figure of 36.8% for pneumococci resistant to erythromycin [17].

The invasive potential of pneumococci is linked not only to serotype but also to genotype [38]–[40]; the isolation of uncharacterised pneumococcal ST from blood CSF and pleural samples in Malaysia highlights the need for further studies especially when considering conjugate vaccine use. Surveillance of antimicrobial resistance, linked to good serotype and genotype data for pneumococci in Malaysia is important in order to determine the potential of any vaccine to reduce resistance rates as well as reducing mortality and morbidity from pneumococcal disease. Collection of genotype data is important in order to monitor the clonal distribution of the pneumococcal population. We and others [41] strongly recommend that further multi-centre studies employing MLST and serotyping to characterise both disease causing and carried pneumococci be performed in Malaysia. Such studies are required to track trends and provide good evidence on which to base policy decisions around the use of PCVs.

## Supporting Information

Figure S1 **The fourteen clonal complexes (CC) into which ST identified in this study were assigned by eBurst (ST taken from the entire** ***S. pneumoniae*** **dataset at mlst.net accessed 30.07.2013).** ST identified in this study are shown by pink circles.). Each black dot represents one ST. Dot sizes represent the number of isolates of a given ST. Lines join STs which share 6 of 7 loci. Blue dots indicate the estimated founders of CCs and yellow dots indicate sub-founders. Figure S1 shows CC199.
(TIF)

Figure S2 **The fourteen clonal complexes (CC) into which ST identified in this study were assigned by eBurst (ST taken from the entire** ***S. pneumoniae*** **dataset at mlst.net accessed 30.07.2013).** ST identified in this study are shown by pink circles.). Each black dot represents one ST. Dot sizes represent the number of isolates of a given ST. Lines join STs which share 6 of 7 loci. Blue dots indicate the estimated founders of CCs and yellow dots indicate sub-founders. Figure S2 shows CC81.
(TIF)

Figure S3 **The fourteen clonal complexes (CC) into which ST identified in this study were assigned by eBurst (ST taken from the entire** ***S. pneumoniae*** **dataset at mlst.net accessed 30.07.2013).** ST identified in this study are shown by pink circles.). Each black dot represents one ST. Dot sizes represent the number of isolates of a given ST. Lines join STs which share 6 of 7 loci. Blue dots indicate the estimated founders of CCs and yellow dots indicate sub-founders. Figure S3 shows CC156.
(TIF)

Figure S4 **The fourteen clonal complexes (CC) into which ST identified in this study were assigned by eBurst (ST taken from the entire** ***S. pneumoniae*** **dataset at mlst.net accessed 30.07.2013).** ST identified in this study are shown by pink circles.). Each black dot represents one ST. Dot sizes represent the number of isolates of a given ST. Lines join STs which share 6 of 7 loci. Blue dots indicate the estimated founders of CCs and yellow dots indicate sub-founders. Figure S4 shows CC473.
(TIF)

Figure S5 **The fourteen clonal complexes (CC) into which ST identified in this study were assigned by eBurst (ST taken from the entire** ***S. pneumoniae*** **dataset at mlst.net accessed 30.07.2013).** ST identified in this study are shown by pink text.). Each black dot represents one ST. Dot sizes represent the number of isolates of a given ST. Lines join STs which share 6 of 7 loci. Blue dots indicate the estimated founders of CCs and yellow dots indicate sub-founders. Figure S5 shows CC62.
(TIF)

Figure S6 **The fourteen clonal complexes (CC) into which ST identified in this study were assigned by eBurst (ST taken from the entire** ***S. pneumoniae*** **dataset at mlst.net accessed 30.07.2013).** ST identified in this study are shown by pink text.). Each black dot represents one ST. Dot sizes represent the number of isolates of a given ST. Lines join STs which share 6 of 7 loci. Blue dots indicate the estimated founders of CCs and yellow dots indicate sub-founders. Figure S6 shows CC113.
(TIF)

Figure S7 **The fourteen clonal complexes (CC) into which ST identified in this study were assigned by eBurst (ST taken from the entire** ***S. pneumoniae*** **dataset at mlst.net accessed 30.07.2013).** ST identified in this study are shown by pink text.). Each black dot represents one ST. Dot sizes represent the number of isolates of a given ST. Lines join STs which share 6 of 7 loci. Blue dots indicate the estimated founders of CCs and yellow dots indicate sub-founders. Figure S7 shows CC221.
(TIF)

Figure S8 **The fourteen clonal complexes (CC) into which ST identified in this study were assigned by eBurst (ST taken from the entire** ***S. pneumoniae*** **dataset at mlst.net accessed 30.07.2013).** ST identified in this study are shown by pink text.). Each black dot represents one ST. Dot sizes represent the number of isolates of a given ST. Lines join STs which share 6 of 7 loci. Blue dots indicate the estimated founders of CCs and yellow dots indicate sub-founders. Figure S8 shows CC320.
(TIF)

Figure S9 **The fourteen clonal complexes (CC) into which ST identified in this study were assigned by eBurst (ST taken from the entire** ***S. pneumoniae*** **dataset at mlst.net accessed 30.07.2013).** ST identified in this study are shown by pink text.). Each black dot represents one ST. Dot sizes represent the number of isolates of a given ST. Lines join STs which share 6 of 7 loci. Blue dots indicate the estimated founders of CCs and yellow dots indicate sub-founders. Figure S9 shows CC2754.
(TIF)

Figure S10 **The fourteen clonal complexes (CC) into which ST identified in this study were assigned by eBurst (ST taken from the entire** ***S. pneumoniae*** **dataset at mlst.net accessed 30.07.2013).** ST identified in this study are shown by pink text.). Each black dot represents one ST. Dot sizes represent the number of isolates of a given ST. Lines join STs which share 6 of 7 loci. Blue dots indicate the estimated founders of CCs and yellow dots indicate sub-founders. Figure S10 shows CC1439.
(TIF)

Figure S11 **The fourteen clonal complexes (CC) into which ST identified in this study were assigned by eBurst (ST taken from the entire** ***S. pneumoniae*** **dataset at mlst.net accessed 30.07.2013).** ST identified in this study are shown by pink text.). Each black dot represents one ST. Dot sizes represent the number of isolates of a given ST. Lines join STs which share 6 of 7 loci. Blue dots indicate the estimated founders of CCs and yellow dots indicate sub-founders. Figure S11 shows CC3784.
(TIF)

Figure S12 **The fourteen clonal complexes (CC) into which ST identified in this study were assigned by eBurst (ST taken from the entire** ***S. pneumoniae*** **dataset at mlst.net accessed 30.07.2013).** ST identified in this study are shown by pink text.). Each black dot represents one ST. Dot sizes represent the number of isolates of a given ST. Lines join STs which share 6 of 7 loci. Blue dots indicate the estimated founders of CCs and yellow dots indicate sub-founders. Figure S12 shows CC180.
(TIF)

Figure S13 **The fourteen clonal complexes (CC) into which ST identified in this study were assigned by eBurst (ST taken from the entire** ***S. pneumoniae*** **dataset at mlst.net accessed 30.07.2013).** ST identified in this study are shown by pink text.). Each black dot represents one ST. Dot sizes represent the number of isolates of a given ST. Lines join STs which share 6 of 7 loci. Blue dots indicate the estimated founders of CCs and yellow dots indicate sub-founders. Figure S13 shows CC5442.
(TIF)

Figure S14 **The fourteen clonal complexes (CC) into which ST identified in this study were assigned by eBurst (ST taken from the entire** ***S. pneumoniae*** **dataset at mlst.net accessed 30.07.2013).** ST identified in this study are shown by pink text.). Each black dot represents one ST. Dot sizes represent the number of isolates of a given ST. Lines join STs which share 6 of 7 loci. Blue dots indicate the estimated founders of CCs and yellow dots indicate sub-founders. Figure S14 shows CC458.
(TIF)
