# Supplementary figures and images for: Novel Clones of Streptococcus pneumoniae Causing Invasive Disease in Malaysia
Source: PLoS One. 2014 Jun 18;9(6):e97912. doi: 10.1371/journal.pone.0097912 (PMC4062404; doi:10.1371/journal.pone.0097912)

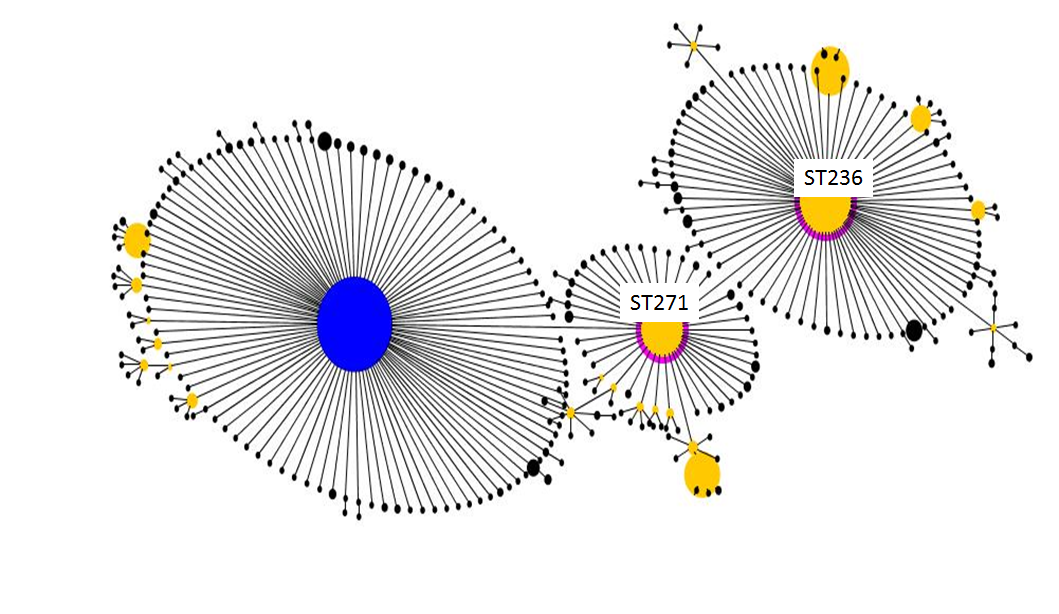

Supplement: Figure S1 — The fourteen clonal complexes (CC) into which ST identified in this study were assigned by eBurst (ST taken from the entire S. pneumoniae dataset at mlst.net accessed 30.07.2013). ST identified in this study are shown by pink circles.). Each black dot represents one ST. Dot sizes represent the number of isolates of a given ST. Lines join STs which share 6 of 7 loci. Blue dots indicate the estimated founders of CCs and yellow dots indicate sub-founders. Figure S1 shows CC199. (TIF) [file pone.0097912.s001.tif]

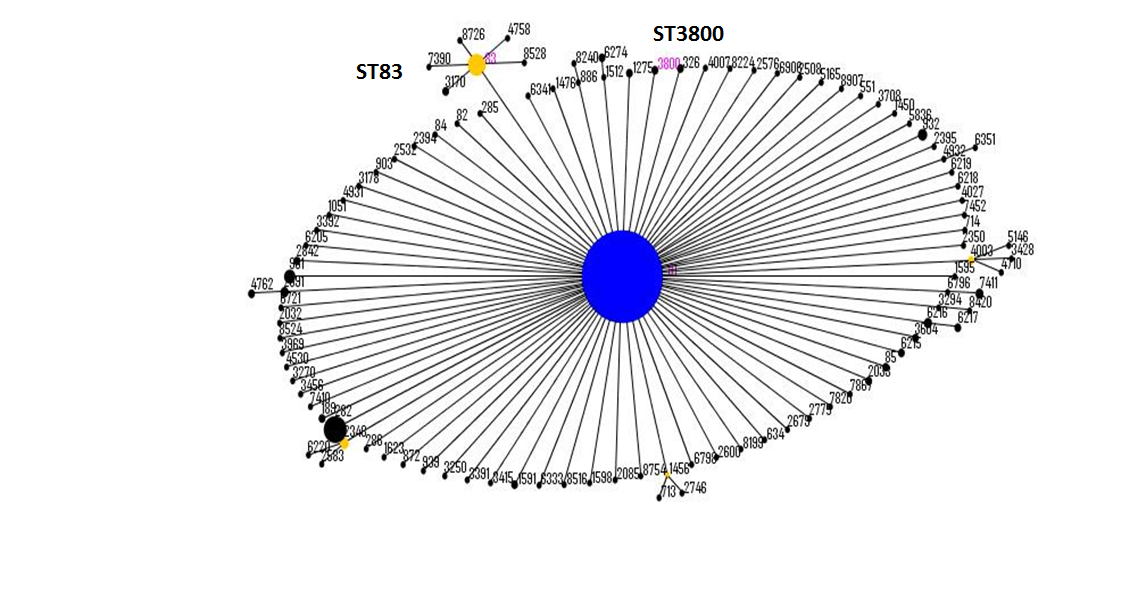

Supplement: Figure S2 — The fourteen clonal complexes (CC) into which ST identified in this study were assigned by eBurst (ST taken from the entire S. pneumoniae dataset at mlst.net accessed 30.07.2013). ST identified in this study are shown by pink circles.). Each black dot represents one ST. Dot sizes represent the number of isolates of a given ST. Lines join STs which share 6 of 7 loci. Blue dots indicate the estimated founders of CCs and yellow dots indicate sub-founders. Figure S2 shows CC81. (TIF) [file pone.0097912.s002.tif]

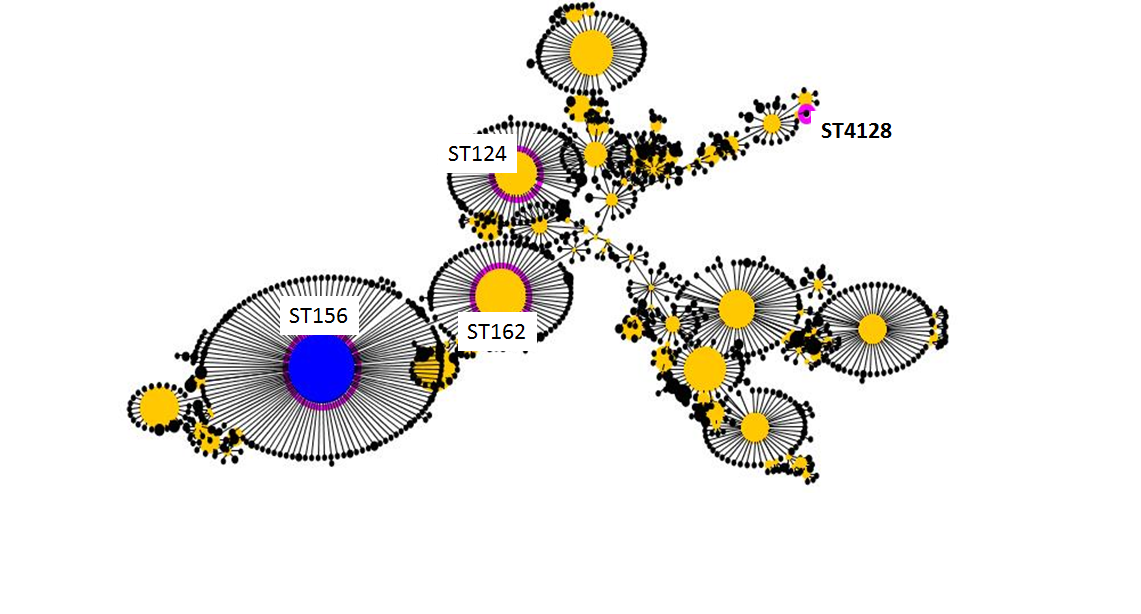

Supplement: Figure S3 — The fourteen clonal complexes (CC) into which ST identified in this study were assigned by eBurst (ST taken from the entire S. pneumoniae dataset at mlst.net accessed 30.07.2013). ST identified in this study are shown by pink circles.). Each black dot represents one ST. Dot sizes represent the number of isolates of a given ST. Lines join STs which share 6 of 7 loci. Blue dots indicate the estimated founders of CCs and yellow dots indicate sub-founders. Figure S3 shows CC156. (TIF) [file pone.0097912.s003.tif]

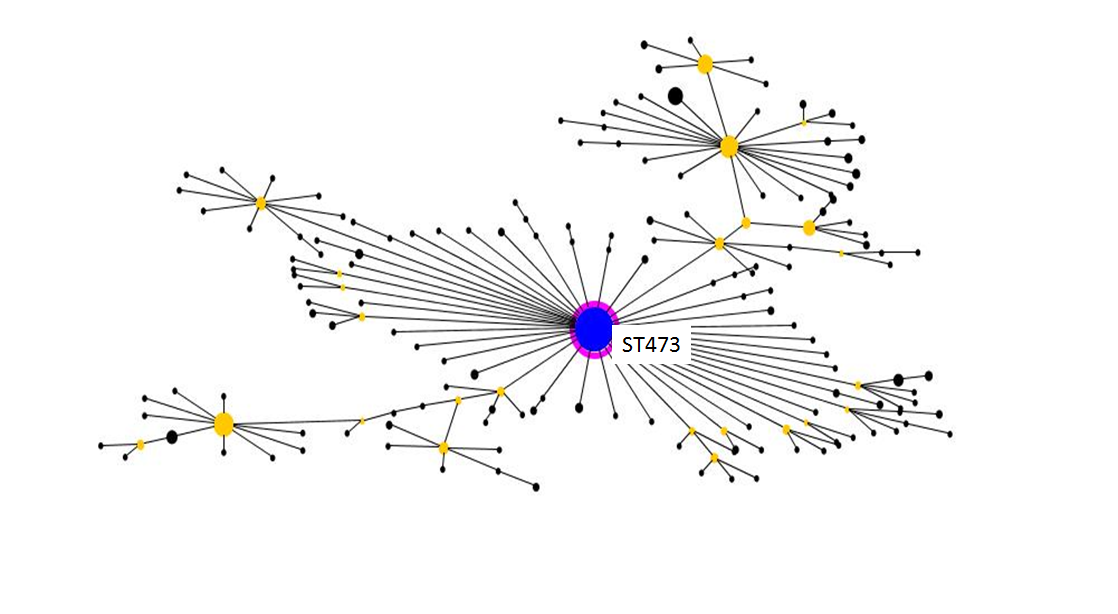

Supplement: Figure S4 — The fourteen clonal complexes (CC) into which ST identified in this study were assigned by eBurst (ST taken from the entire S. pneumoniae dataset at mlst.net accessed 30.07.2013). ST identified in this study are shown by pink circles.). Each black dot represents one ST. Dot sizes represent the number of isolates of a given ST. Lines join STs which share 6 of 7 loci. Blue dots indicate the estimated founders of CCs and yellow dots indicate sub-founders. Figure S4 shows CC473. (TIF) [file pone.0097912.s004.tif]

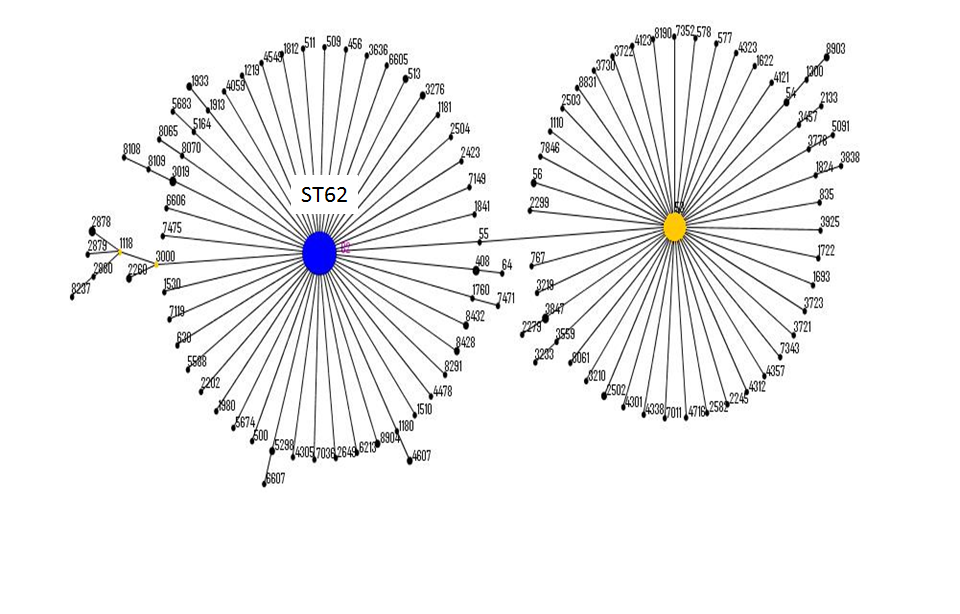

Supplement: Figure S5 — The fourteen clonal complexes (CC) into which ST identified in this study were assigned by eBurst (ST taken from the entire S. pneumoniae dataset at mlst.net accessed 30.07.2013). ST identified in this study are shown by pink text.). Each black dot represents one ST. Dot sizes represent the number of isolates of a given ST. Lines join STs which share 6 of 7 loci. Blue dots indicate the estimated founders of CCs and yellow dots indicate sub-founders. Figure S5 shows CC62. (TIF) [file pone.0097912.s005.tif]

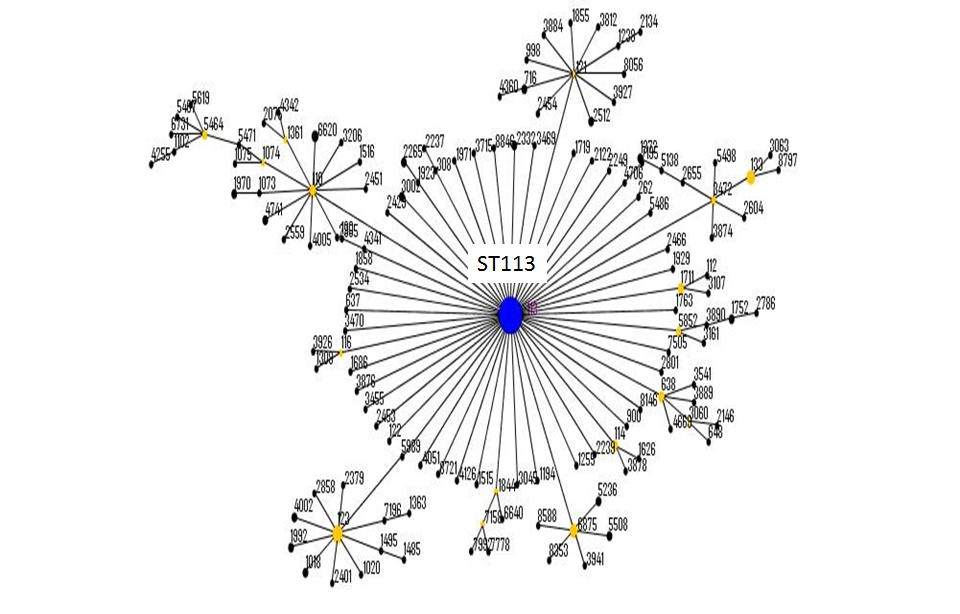

Supplement: Figure S6 — The fourteen clonal complexes (CC) into which ST identified in this study were assigned by eBurst (ST taken from the entire S. pneumoniae dataset at mlst.net accessed 30.07.2013). ST identified in this study are shown by pink text.). Each black dot represents one ST. Dot sizes represent the number of isolates of a given ST. Lines join STs which share 6 of 7 loci. Blue dots indicate the estimated founders of CCs and yellow dots indicate sub-founders. Figure S6 shows CC113. (TIF) [file pone.0097912.s006.tif]

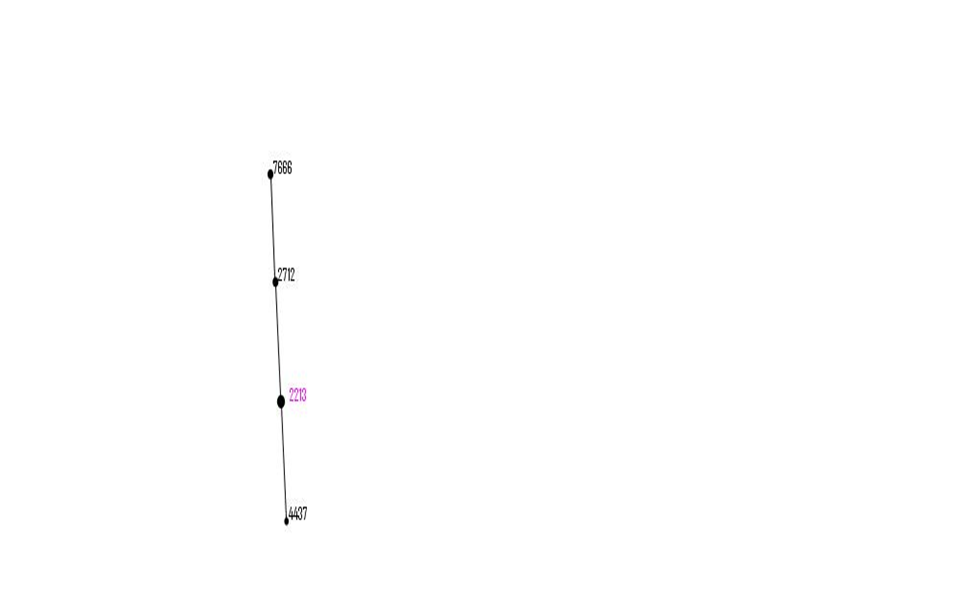

Supplement: Figure S7 — The fourteen clonal complexes (CC) into which ST identified in this study were assigned by eBurst (ST taken from the entire S. pneumoniae dataset at mlst.net accessed 30.07.2013). ST identified in this study are shown by pink text.). Each black dot represents one ST. Dot sizes represent the number of isolates of a given ST. Lines join STs which share 6 of 7 loci. Blue dots indicate the estimated founders of CCs and yellow dots indicate sub-founders. Figure S7 shows CC221. (TIF) [file pone.0097912.s007.tif]

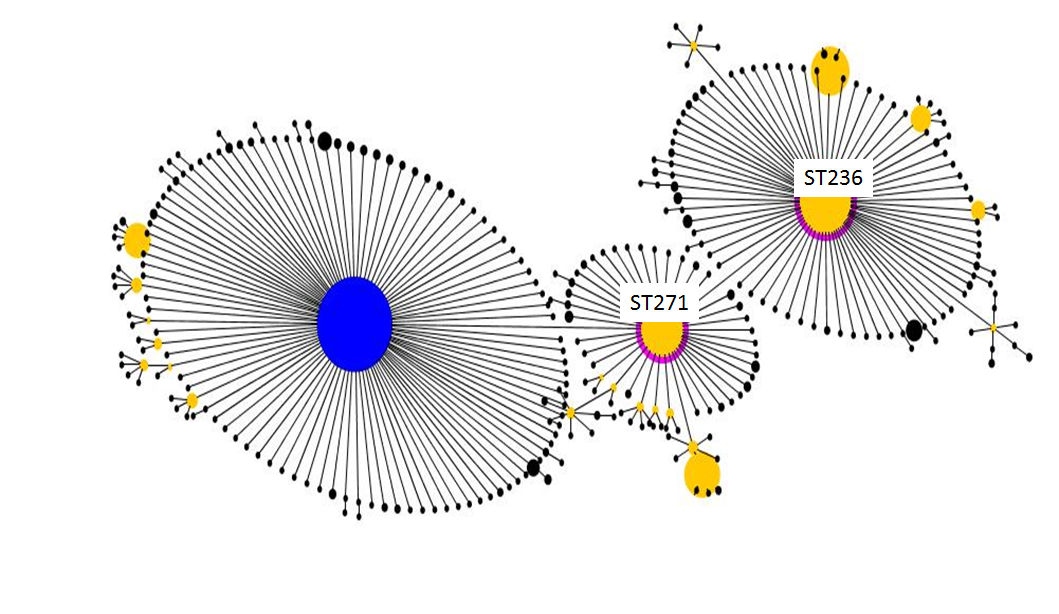

Supplement: Figure S8 — The fourteen clonal complexes (CC) into which ST identified in this study were assigned by eBurst (ST taken from the entire S. pneumoniae dataset at mlst.net accessed 30.07.2013). ST identified in this study are shown by pink text.). Each black dot represents one ST. Dot sizes represent the number of isolates of a given ST. Lines join STs which share 6 of 7 loci. Blue dots indicate the estimated founders of CCs and yellow dots indicate sub-founders. Figure S8 shows CC320. (TIF) [file pone.0097912.s008.tif]

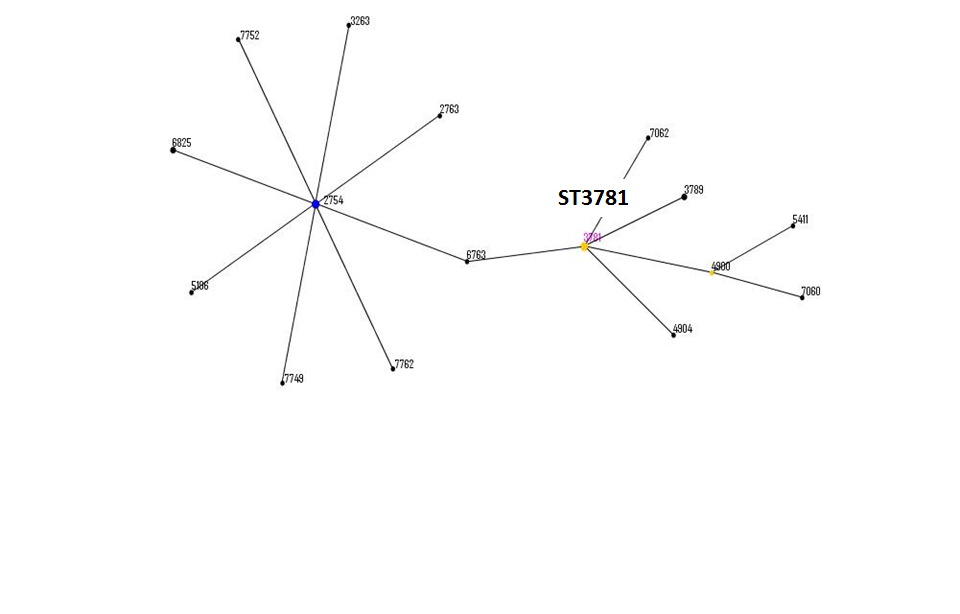

Supplement: Figure S9 — The fourteen clonal complexes (CC) into which ST identified in this study were assigned by eBurst (ST taken from the entire S. pneumoniae dataset at mlst.net accessed 30.07.2013). ST identified in this study are shown by pink text.). Each black dot represents one ST. Dot sizes represent the number of isolates of a given ST. Lines join STs which share 6 of 7 loci. Blue dots indicate the estimated founders of CCs and yellow dots indicate sub-founders. Figure S9 shows CC2754. (TIF) [file pone.0097912.s009.tif]

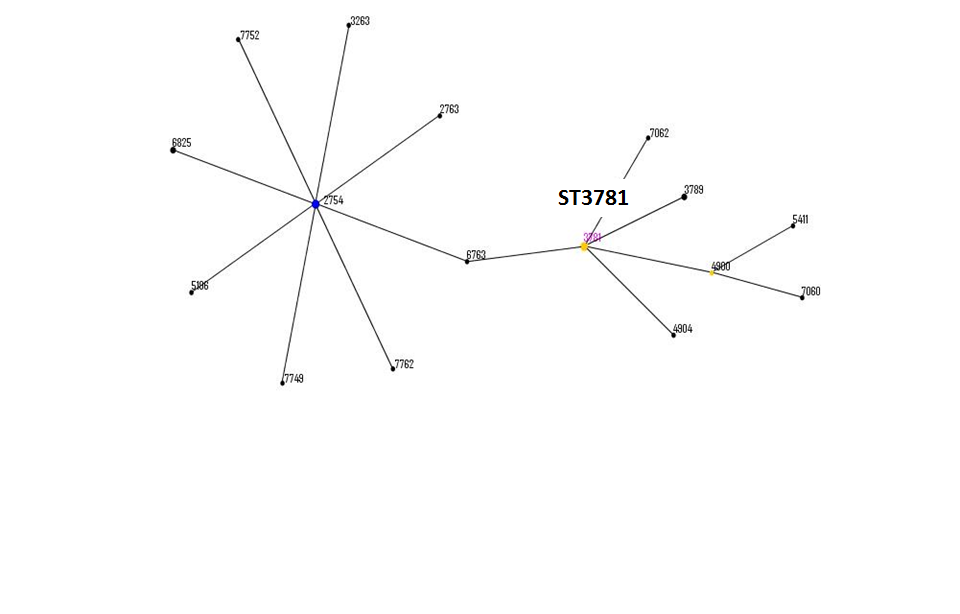

Supplement: Figure S10 — The fourteen clonal complexes (CC) into which ST identified in this study were assigned by eBurst (ST taken from the entire S. pneumoniae dataset at mlst.net accessed 30.07.2013). ST identified in this study are shown by pink text.). Each black dot represents one ST. Dot sizes represent the number of isolates of a given ST. Lines join STs which share 6 of 7 loci. Blue dots indicate the estimated founders of CCs and yellow dots indicate sub-founders. Figure S10 shows CC1439. (TIF) [file pone.0097912.s010.tif]

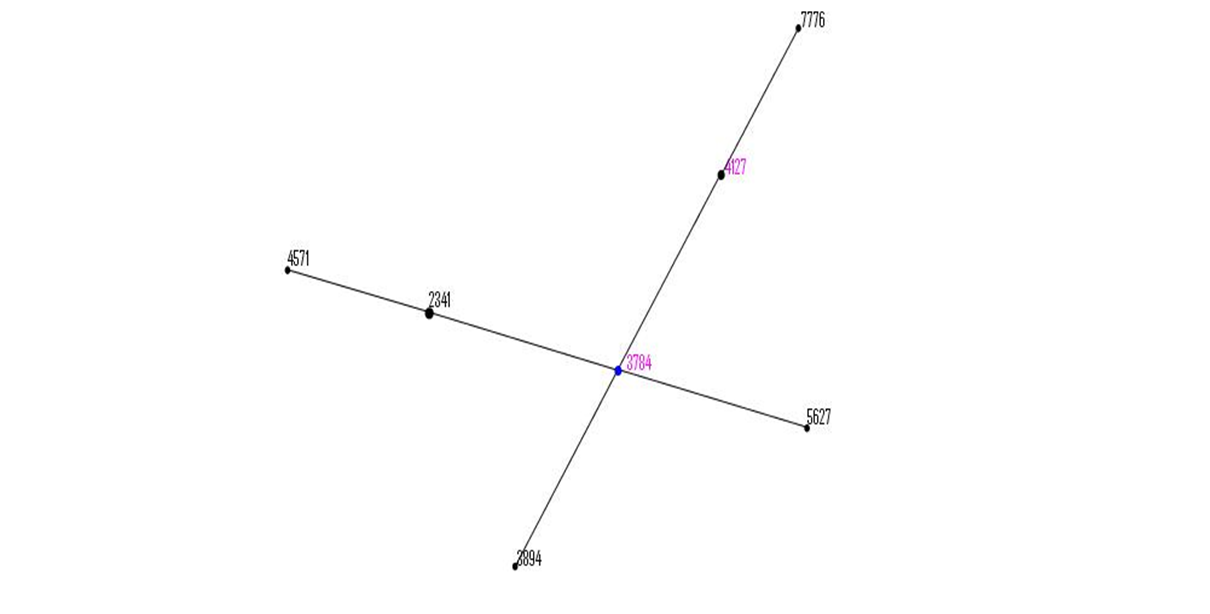

Supplement: Figure S11 — The fourteen clonal complexes (CC) into which ST identified in this study were assigned by eBurst (ST taken from the entire S. pneumoniae dataset at mlst.net accessed 30.07.2013). ST identified in this study are shown by pink text.). Each black dot represents one ST. Dot sizes represent the number of isolates of a given ST. Lines join STs which share 6 of 7 loci. Blue dots indicate the estimated founders of CCs and yellow dots indicate sub-founders. Figure S11 shows CC3784. (TIF) [file pone.0097912.s011.tif]

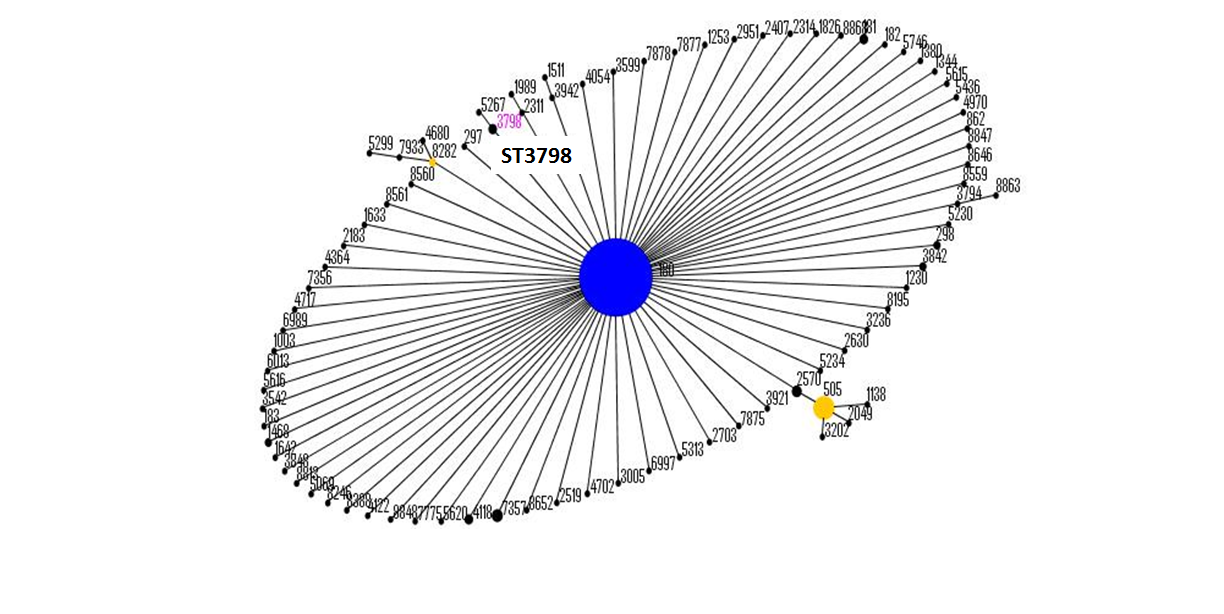

Supplement: Figure S12 — The fourteen clonal complexes (CC) into which ST identified in this study were assigned by eBurst (ST taken from the entire S. pneumoniae dataset at mlst.net accessed 30.07.2013). ST identified in this study are shown by pink text.). Each black dot represents one ST. Dot sizes represent the number of isolates of a given ST. Lines join STs which share 6 of 7 loci. Blue dots indicate the estimated founders of CCs and yellow dots indicate sub-founders. Figure S12 shows CC180. (TIF) [file pone.0097912.s012.tif]

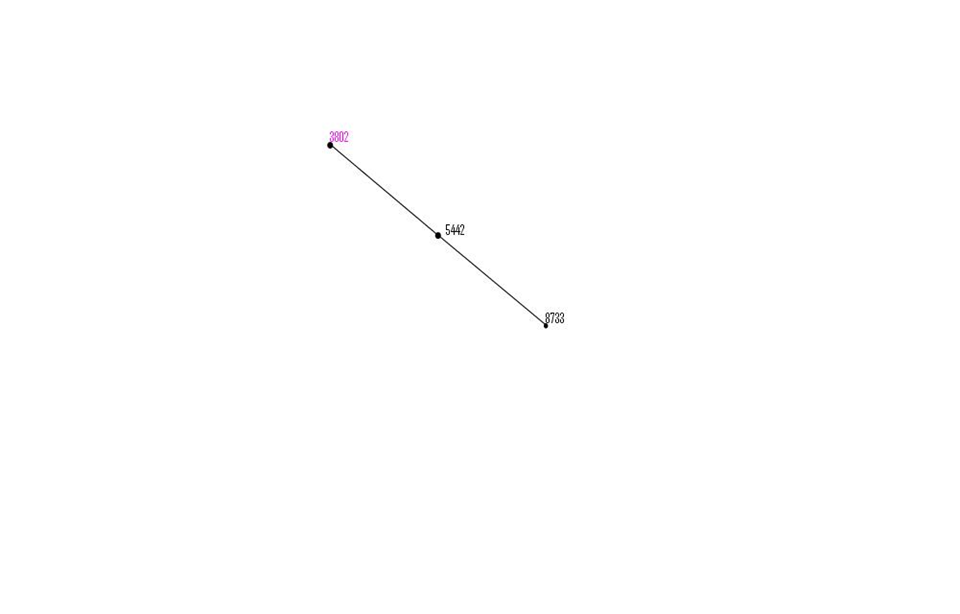

Supplement: Figure S13 — The fourteen clonal complexes (CC) into which ST identified in this study were assigned by eBurst (ST taken from the entire S. pneumoniae dataset at mlst.net accessed 30.07.2013). ST identified in this study are shown by pink text.). Each black dot represents one ST. Dot sizes represent the number of isolates of a given ST. Lines join STs which share 6 of 7 loci. Blue dots indicate the estimated founders of CCs and yellow dots indicate sub-founders. Figure S13 shows CC5442. (TIF) [file pone.0097912.s013.tif]

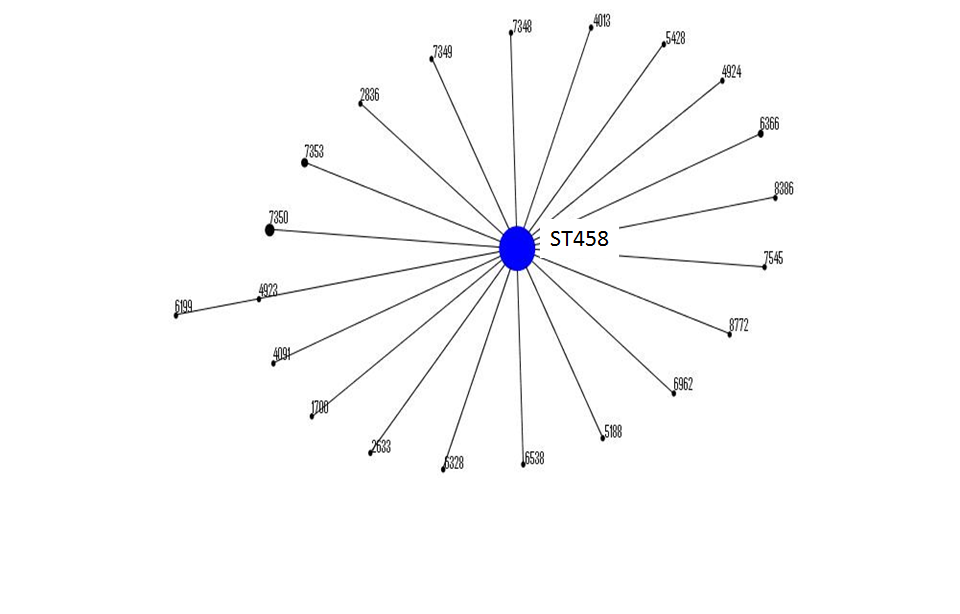

Supplement: Figure S14 — The fourteen clonal complexes (CC) into which ST identified in this study were assigned by eBurst (ST taken from the entire S. pneumoniae dataset at mlst.net accessed 30.07.2013). ST identified in this study are shown by pink text.). Each black dot represents one ST. Dot sizes represent the number of isolates of a given ST. Lines join STs which share 6 of 7 loci. Blue dots indicate the estimated founders of CCs and yellow dots indicate sub-founders. Figure S14 shows CC458. (TIF) [file pone.0097912.s014.tif]
